# Arbeitsbedingte Fatigue in der Anästhesie und Intensivmedizin: Übersichtsarbeit zu einem strukturellen Problem

**DOI:** 10.1007/s00101-025-01622-6

**Published:** 2026-01-19

**Authors:** Gerrit Herpertz, Friederike Roesch, Igor Abramovich, Alexandra Trinks, Verena Ghezel-Ahmadi, Martina Nowak-Machen, Karin Becke-Jakob

**Affiliations:** 1 Universitätsklinik für Anästhesiologie, Intensivmedizin, Notfallmedizin und Schmerztherapie, Klinikum Oldenburg AöR, Rahel-Straus-Straße 10, 26133 Oldenburg, Deutschland; 2 Fakultät VI Medizin und Gesundheitswissenschaften, Carl von Ossietzky Universität Oldenburg, Oldenburg, Deutschland; 3 Klinik für Anästhesiologie, Intensivmedizin, Notfallmedizin und Schmerztherapie, Klinikum Ludwigsburg, Ludwigsburg, Deutschland; 4 Department of Anesthesia and Perioperative Care, University of California, San Francisco, CA USA; 5 Klinik für Anästhesiologie, Klinikum der Universität München (LMU), München, Deutschland; 6 Klinik für Anästhesie, Operative Intensivmedizin und Schmerztherapie, Universitätsklinikum Mannheim GmbH, Mannheim, Deutschland; 7 Klinik für Anästhesie und Intensivmedizin, Palliativ- und Schmerzmedizin, Klinikum Ingolstadt, Ingolstadt, Deutschland; 8 Anästhesiologie, Universitäts-Kinderspital beider Basel UKBB, Basel, Schweiz

**Keywords:** Human Ressources, Burn-out, Prävention, Patientensicherheit, Schichtarbeit, Human Ressources, Burnout, Prevention, Patient safety, Shift work

## Abstract

Arbeitsbedingte Fatigue ist ein ernst zu nehmendes psychophysiologisches Phänomen, das sich durch Erschöpfung, Konzentrationsprobleme, verminderte Wachsamkeit sowie eingeschränkte Entscheidungsfähigkeit äußert. Häufig entsteht sie durch gestörte Schlafmuster und Schichtarbeit und erhöht das Risiko kritischer Zwischenfälle im Klinikalltag. Besonders betroffen sind Anästhesist:innen, deren unregelmäßige Arbeitszeiten den zirkadianen Rhythmus stören. Obwohl Fatigue reversibel ist, wenn geeignete Maßnahmen ergriffen werden, wird sie in Deutschland bislang kaum als strukturelles Problem erkannt.

In den letzten Jahrzehnten haben sich die Arbeitsbedingungen im europäischen Gesundheitswesen zunehmend verschlechtert – ein Trend, der in der COVID-19-Pandemie seinen Höhepunkt fand. Diese Entwicklung hat gezeigt, dass das Wohlergehen medizinischer Fachkräfte stärker in den Fokus rücken muss. Die vorliegende Übersichtsarbeit will das Bewusstsein für Fatigue schärfen und Einblicke in wirksame Managementstrategien geben. Dabei werden sowohl internationale Konzepte als auch lokale Lösungsansätze für das deutsche System betrachtet.

Die nun vorliegende Übersicht und Analyse stützt sich auf Studien und Materialien aus dem europäischen „Fatigue Project“ sowie der „Fight-Fatigue“-Kampagne. Sie beleuchtet die Auswirkungen von Fatigue über alle Karrierephasen hinweg und identifiziert praxisnahe Maßnahmen zur Risikoreduktion.

Die Ergebnisse verdeutlichen, dass Fatigue alle Berufsphasen betrifft. Strukturiertes Fatigue-Management ist daher ein zentraler Bestandteil nachhaltiger Gesundheitsversorgung. Besonders wirksam zeigen sich Fatigue-Risk-Management-Systeme und eine optimierte Dienstplanung, um die Belastung des Personals zu reduzieren und die Patientensicherheit zu erhöhen.

## Abstract (en)

Work-related fatigue is a serious psychophysiological phenomenon characterized by exhaustion, impaired concentration, reduced alertness and diminished decision-making capacity. It often results from disrupted sleep patterns and shift work and increases the risk of critical incidents in the clinical practice. Anesthetists are particularly affected as irregular working hours and frequent night shifts disrupt their circadian rhythms. Although fatigue is reversible with appropriate measures, it remains largely unrecognized as a structural issue within the German healthcare system.

Over recent decades the working conditions across European healthcare settings have steadily deteriorated, a trend that culminated during the COVID-19 pandemic. This period clearly highlighted the urgent need to prioritize the well-being of healthcare professionals. The aim of this review article is to raise awareness of fatigue and provide insights into effective management strategies. It explores both international concepts and local solutions relevant to the German system.

This review and analysis are based on studies and material developed as part of the European “Fatigue Project” and the “Fight Fatigue” campaign. It examines the effects of fatigue across all career stages and identifies practical strategies for risk reduction.

The results show that fatigue affects anesthetists at all stages of their careers. Structured fatigue management is therefore a vital component of sustainable healthcare provision. In particular, fatigue risk management systems and optimized shift work planning have proven effective in reducing the burden on personnel and enhancing patient safety.

Arbeitsbedingte Fatigue, auch als „Erschöpfungssyndrom“ bekannt, ist ein Problem, das viele Kolleg:innen in der Anästhesiologie regelmäßig erleben. Dieses Syndrom ist nicht spezifisch für die Gesundheitsbranche, sondern tritt auch in anderen sicherheitskritischen Bereichen auf. Besonders die Luftfahrt, aus der wir bereits das Konzept des „Crew Resource Management“ adaptiert haben, hat das Problem erkannt und entsprechende Strategien sowie Lösungsansätze entwickelt [24]. Auch andere risikobehaftete Sektoren wie Speditionswesen, Bahnverkehr und Atomindustrie haben Kontrollmechanismen eingeführt, um Fatigue-bedingte Zwischenfälle zu vermeiden. Im deutschen Gesundheitswesen fehlen jedoch bislang vergleichbare Sicherheitsstrukturen [47].

## Hintergrund

**Abb. 1:**
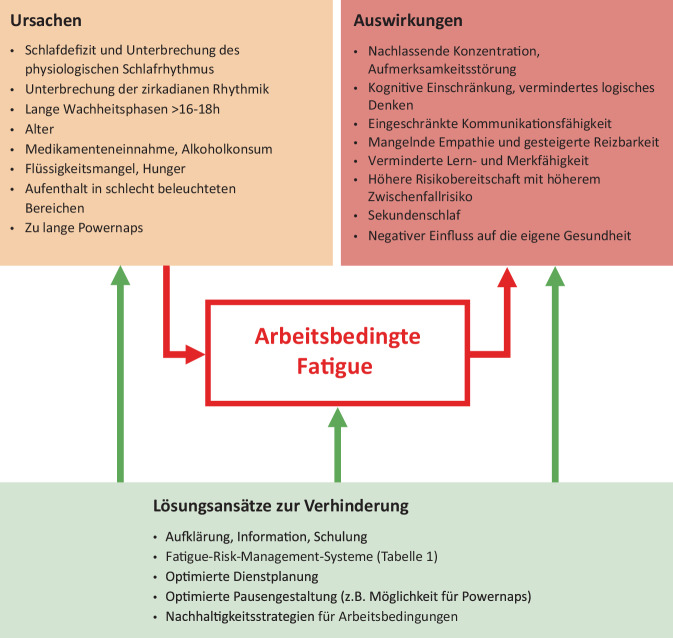
Arbeitsbedingte Fatigue-Ursachen, Auswirkungen und Lösungsansätze

Arbeitsbedingte Fatigue („Erschöpfungssyndrom“) ist gekennzeichnet durch Müdigkeit, Erschöpfung, verminderte Leistungsfähigkeit, Konzentrationsverlust und erhöhte Fehleranfälligkeit; eine Thematik, die viele Kolleg:innen – insbesondere im Nachtdienst – regelmäßig erleben. Vielfältige Faktoren begünstigen die Entstehung von Fatigue und sie wirkt sich sowohl auf uns persönlich als auch auf unsere Patient:innen aus Tab. 1 (Abb. 1). Allen voran steht die Unterbrechung des normalen Schlafverhaltens und des zirkadianen Rhythmus. Beeinflusst wird beides insbesondere durch Tageslicht [47]. Es liegt in der Natur des ärztlichen Berufes, dass eine Versorgung rund um die Uhr gewährleistet wird. Somit sind Anästhesist:innen zwangsläufig durch die Arbeitsbedingungen einem erhöhten Risiko für Fatigue ausgesetzt.

**Tab. 1:** Lösungsansätze

Lösungsansatz	Beispiel	Effekt
Fatigue-Risk-Management-System (FRMS)	Fatigue-Meldungen durch Arbeitnehmer:innen	Identifikation von Fatigue-Risiken
	Nutzung von Dienstplanungssoftware	Fatigue-Prävention
Pausenräume und Powernaps	Verpflichtende Bereitstellung von Pausenräumen	Fatigue-Prävention durch ausgeruhte Mitarbeiter:innen
Schulung zur Nachtarbeit	Einführung von Schlafhygiene und individuelle Einflussnahme auf das Fatigue-Risiko	Erkennen individueller Einflussfaktoren
		Erlernen von Coping-Strategien
Arbeitsentlastung	Limitierung der Arbeit auf notwendige Fälle und Notfälle	Gesteigerte Patientensicherheit durch weniger Zwischenfälle
	Kein Elektivprogramm in der Nacht	Entlastung der Mitarbeitenden, die dann ausgeruht für die wirklichen Notfälle sind

Neben der individuellen Gefährdung, beispielsweise auf dem Heimweg, stellt Fatigue auch ein nichterhebliches Sicherheitsrisiko für Patient:innen dar. Das European Board of Anaesthesiology (EBA) und insbesondere das Workforce, Working condition, Welfare Standing Committee (WWW; www. https://eba-uems.eu/standing-committees/www) beschäftigen sich seit 2019 mit dem „Fatigue Project“. Dort werden europaweit Daten erhoben, um ein Bewusstsein für das Problem zu schaffen und langfristig ein Umdenken in der Arbeitskultur der Anästhesie und Intensivmedizin zu fördern [22]. Im Vereinigten Königreich wurde das Thema bereits 2016 aufgegriffen; Auslöser war der tragische Unfalltod eines Weiterbildungsassistenten nach einem Nachtdienst [14]. Es wäre mehr als tragisch, wenn auch in Deutschland erst ein vermeidbarer Todesfall notwendig wäre, um diesem Thema die erforderliche Aufmerksamkeit zu verschaffen.

Fatigue ist nicht nur ein individuelles Problem – es ist ein strukturelles Defizit im Gesundheitswesen

Die Kenntnis von Fatigue als oft latentes und schleichendes Problem und seiner Ursachen ist der erste wesentliche Schritt, um potenziellen Patient:innenschäden vorzubeugen und das Wohlbefinden der Kolleg:innen zu verbessern. Die Autor:innen dieses Artikels unterstützen die Positionen des WWW und möchten konkret auf das Problem der Fatigue in der Anästhesiologie und Intensivmedizin sowie dessen Auswirkungen aufmerksam machen.

## Arbeitsbedingte Fatigue in verschiedenen Karrierephasen in der Anästhesiologie

### Weiterbildung Anästhesiologie

Besonders Ärzt:innen in der Weiterbildung (ÄiW) verdienen bei der Bewertung der Fatigue-Risiken besondere Aufmerksamkeit [17]. Sie spielen eine zentrale Rolle in der nächtlichen Patientenversorgung, sind hohen klinischen Arbeitsbelastungen ausgesetzt, erleben den Druck ihrer Facharztweiterbildung und müssen oft die Doppelbelastung von Beruf und junger Familie bewältigen.

Die meisten ÄiW in Deutschland haben durchschnittlich 4 bis 6 Nachtdienste/Monat. Aufgrund der häufigen Nachtarbeit und des ständigen Schichtwechsels sind sie besonders anfällig für die negativen Auswirkungen von arbeitsbedingter Fatigue [3, 6]. Die Schlafqualität leidet auch an den Erholungstagen zwischen den Nachtdienstblöcken, was zu einer kumulativen Erschöpfung führen kann [3].

Ziel der Weiterbildungszeit ist, durch Fortbildung, das Erlernen technischer Fähigkeiten und Eigenstudium eine hohe Expertise in der Anästhesiologie zu entwickeln. Arbeitsbedingte Fatigue jedoch schränkt die kognitive Leistungsfähigkeit ein und verringert sowohl die Lernfähigkeit als auch die Fähigkeit, sich effektiv auf Prüfungen vorzubereiten [4]. Dies stellt eine Herausforderung dar, da die Weiterbildung eine Phase intensiven Lernens und beruflicher Entwicklung ist und zugleich das Fundament für die zukünftige Generation von Anästhesist:innen bildet. Fatigue erhöht das Fehlerpotenzial, was durch die geringere Erfahrung und fehlende Coping-Strategien der ÄiW noch verstärkt wird. Mehr als die Hälfte der in einer deutschen Studie befragten ÄiW gaben an, bereits Fehler oder Beinahe-Fehler aufgrund von Ermüdung gemacht zu haben [3]. Dies umfasst Fehler bei der klinischen Entscheidungsfindung, Kommunikationsprobleme, Schwierigkeiten bei Verfahren sowie Medikamenten- und Verschreibungsfehler [2].

In Nachtdiensten sollten Pausen fester Bestandteil und die Verschiebung von Tätigkeiten möglich sein

Früh einsetzende Erschöpfung kann dazu führen, dass ÄiW den klinischen Alltag früher als geplant verlassen oder ein Burn-out erleiden [38]. Etwa 50 % der ÄiW erwägen eine Reduktion der Arbeitszeit [9]. Langfristige Auswirkungen von Fatigue bei ÄiW zeigen sich beispielsweise in Großbritannien, wo etwa 70 % der Befragten Auswirkungen auf ihre körperliche und mentale Gesundheit beklagen. Besonders mangelnde Pausen und fehlende Pausenräume während der Nachtarbeit wurden als wesentliche Trigger für Fatigue identifiziert [37].

Ähnliche Ergebnisse zeigen Studien in Deutschland: ÄiW nutzen selten Pausenräume während ihrer Nachtdienste und berichten einen moderat bis stark negativen Einfluss der Erschöpfung auf nahezu alle Lebensbereiche, wie emotionales Wohlbefinden und die Fähigkeit, Hobbys oder Reisen zu genießen oder zu lernen [3]. Knapp ein Drittel der ÄiW schafft es nicht, eine durchgehende 30-minütige Pause während der Arbeit zu nehmen, und bei etwa einem Viertel werden Pausen von Vorgesetzten nicht unterstützt oder untersagt [3]. Die Erwartungen von Vorgesetzten prägen das Verhalten des Nachwuchspersonals und führen zu einer Kluft zwischen der aus Führungsperspektive „vorgestellten“ und der erbrachten „tatsächlichen Arbeit“ durch die AiW. Wenn Führungskräfte trotz offensichtlicher Müdigkeit oder Belastungen durch Nachtarbeit ununterbrochene Leistung fordern, wird die Wahrscheinlichkeit, dass Pausen eingelegt werden, verringert – mit negativen Folgen [44].

Dies würde dazu beitragen, die langfristigen negativen Auswirkungen von arbeitsbedingter Fatigue zu reduzieren, die Gesundheit und Leistungsfähigkeit der ÄiW zu erhalten, einem drohenden Personalmangel in der Anästhesiologie vorzubeugen und das Fach attraktiv für zukünftige Generationen aufzustellen [40].

## Berufsphasen nach der Facharztweiterbildung Anästhesiologie

Mit zunehmender Berufserfahrung steigt die Komplexität der Aufgaben für Anästhesist:innen. Neben anspruchsvollen operativen Eingriffen und Intensivstationseinsätzen übernehmen sie zunehmend organisatorische Tätigkeiten wie Personaleinsatzplanung, Supervision und Priorisierung von Notfalleingriffen im Dienstbetrieb.

Fach- und Oberärzt:innen werden häufig für Hintergrundrufdienste eingeteilt, um bei Rückfragen oder komplexen Fällen verfügbar zu sein. [3]. In gängigen Tarifverträgen (z. B. TV-VKA Ärzte, TV-Ärzte (TdL)) ist eine Obergrenze von 13 Rufdiensten oder aber keine Höchstgrenze festgelegt, und es ist keine Seltenheit, dass Kolleg:innen ganze Wochenenden von Freitag bis Sonntag am Stück übernehmen [3, 49, 50]. Laut einer europäischen Umfrage verließen 51 % der befragten Fachärzt:innen in Deutschland während des Rufdienstes die Klinik nicht, während zwei Drittel während der Schlafenszeit zwei oder mehr Anrufe erhielten und oft in die Klinik kommen mussten. Dies stört den Schlaf erheblich und erhöht das Risiko für Fatigue [3].

Spät- und Nachtdienste erhöhen das Risiko für chronische Fatigue mit typischen Symptomen

Mit zunehmendem Alter verändert sich der Schlaf und wird störanfälliger: Die Einschlafzeit verlängert sich, der Schlaf wird kürzer und flacher, und Tiefschlafphasen nehmen ab [36]. Nachtarbeit bzw. daraus resultierender Tagschlaf beeinträchtigen zusätzlich die Schlafarchitektur und können neuronale Alterung beschleunigen. Schlafmangel und gestörte zirkadiane Rhythmen begünstigen chronische Erkrankungen wie Bluthochdruck, Diabetes, Adipositas und sogar Krebs. Auch die Anfälligkeit für Infekte steigt [4, 39]. Bei über 60-Jährigen sind kognitive Veränderungen wie ein nachlassendes Arbeits- und episodisches Gedächtnis sowie eine langsamere Informationsverarbeitung typisch, die jedoch teilweise durch Erfahrung kompensiert werden können [41].

Infolgedessen kann es zum Burn-out kommen, wenn dieser Teufelskreis nicht durchbrochen wird [15, 29].

Chronische Erkrankungen, Fatigue- und Burn-out-bedingte Ausfälle hochspezialisierter Fachkräfte haben gravierende individuelle und wirtschaftliche Konsequenzen [15, 29, 30, 33, 48].

Maßnahmen gegen Fatigue sollten umgesetzt werden, um auch im Alter den Arbeitsalltag zu bewältigen

**Abb. 2:**
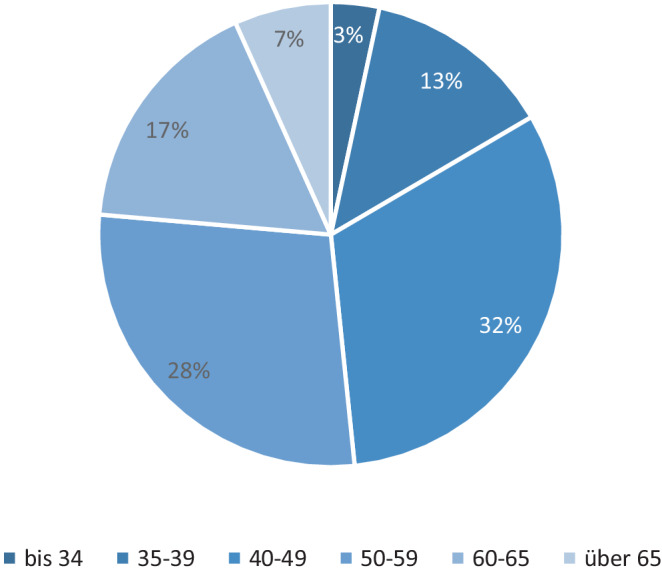
Altersverteilung deutscher Anästhesist:innen 2023 [12]

Angesichts einer alternden Ärzteschaft –über 50 % der Anästhesist:innen in Deutschland sind älter als 50 Jahre. (Abb. 2) – und einer verlängerten Lebensarbeitszeit sollten Aspekte wie Fatigue-Resilienz und Dienstplanung stärker berücksichtigt werden [19]. Bereits 2015 waren 43 % der über 50-Jährigen und 57 % der über 55-Jährigen in Teilzeit tätig, wobei Letztere meist von Bereitschafts- und Wochenenddiensten ausgenommen wurden [32].

## Einfluss von Fatigue auf die Patienten:innensicherheit in der Anästhesiologie

Die arbeitsbedingte Fatigue betrifft nicht nur die Anästhesist:innen selbst, sondern auch die Patient:innen. Während der Nachtarbeit steigt das Risiko für Zwischenfälle um den Faktor 1,44 [16]. Eine Metaanalyse von Cortegiani et al., basierend auf Daten von fast 3 Mio. Patient:innen, zeigt, dass die postoperative Mortalität im Bereitschaftsdienst um den Faktor 1,16 erhöht ist – unabhängig davon, ob der Fokus speziell auf die Anästhesie gelegt wird [18].

In einer Befragung gaben 74 % der Anästhesist:innen an, dass sie ihre nächtliche Fatigue als Risiko für die Patientenversorgung wahrnehmen. Noch mehr Befragte bewerteten die nächtliche Versorgung generell als risikobehaftet [17]. Eine weitere Umfrage ergab, dass 44 % der Anästhesist:innen bereits Fatigue-bedingte Fehler gemacht haben, und bei 3 % der Befragten führte dies mindestens einmal zu einer Schädigung von Patient:innen [46].

Diese Risiken lassen sich auch objektiv belegen: Fehler bei der Verabreichung und Dosierung von Medikamenten treten häufiger auf, kritische Kreislaufsituationen werden mitunter zu spät erkannt, und die Kommunikation im Team ist eingeschränkt [8].

## Reduktion von Fatigue als Teil einer Nachhaltigkeitsstrategie

Eine Nachhaltigkeitsstrategie in der Anästhesiologie umfasst mehr als die Reduktion des CO_2_-Fußabdrucks der Abteilungen. Das Consensus Paper der European Society of Anaesthesiology and Intensive Care (ESAIC) hebt hervor, dass Nachhaltigkeit auch die langfristige Erhaltung des Personals einschließt [27]: „Fachkräfte im Gesundheitswesen sind Eckpfeiler einer nachhaltigen Medizin“. Es werden klare Empfehlungen zur Förderung von Well-Being, Prävention von Burn-out und Erhaltung der psychischen Gesundheit ausgesprochen. Diese Aspekte sind essenziell für die Qualität der Patientenversorgung und sollten von politischen sowie klinischen Entscheidungsträgern anerkannt werden.

Eine europäische Umfrage schlägt konkrete Maßnahmen zur Verbesserung der Arbeitsbedingungen vor [13]: Begrenzung der täglichen Arbeitszeit, Reduktion von ökonomischem Druck, Supervision während der Weiterbildung, ununterbrochene Pausen, Entlastung bei Nachtarbeit und zusätzliche Kompensationszeiten bei hoher Arbeitslast. Zudem sollten alternative Dienstplan- und Arbeitszeitmodelle entwickelt werden, die den Bedürfnissen anästhesiologischer Fachkräfte besser gerecht werden [26].

Folgende Maßnahmen sind wesentliche Bestandteil nachhaltiger Arbeitsbedingungen [27]:

- *Leichte Verfügbarkeit von Unterstützung*: Professionelle Hilfe und Zweitmeinungen von Kolleg:innen sollten während der Nachtarbeit in der Anästhesie und Intensivpflege jederzeit verfügbar sein.
- *Zugang zu Ruheeinrichtungen:* Die regelmäßige Nutzung von Ruhebereichen während und nach der Schicht sollte gefördert und unkompliziert möglich sein.
- *Schulungen zu Müdigkeit:* Ärzt:innen sollten vor Beginn von Nachtarbeit Schulungen zu den Auswirkungen von Müdigkeit auf Leistung und Privatleben, den damit verbundenen Risiken, Schlafhygiene, Ernährung und den rechtlichen Folgen des Fahrens bei Übermüdung erhalten.
- *Psychologische Unterstützung:* Der Zugang zu psychologischer Unterstützung sowie Crisis Care Management, einschließlich Debriefing nach kritischen Ereignissen, sollte niedrigschwellig gewährleistet sein.
- *Anerkennung der Besonderheiten von Nachtarbeit:* Nachtarbeit sollte offen als Tätigkeit mit spezifischen Risiken und besonderen Auswirkungen auf das Berufs- und Privatleben anerkannt werden.
- *Einhaltung rechtlicher Vorgaben:* Dienstpläne müssen mit der Europäischen Arbeitszeitrichtlinie (EWTD) oder nationalen Vorschriften (für Länder außerhalb der EU) übereinstimmen.
- *Gesunde Ernährung:* Ein Zugang zu ausgewogener und gesunder Ernährung sollte sichergestellt werden.
- *Körperliche Bewegung:* Hier bieten sich z. B. geförderte Sportprogramme oder ein „Job-Rad“ im Rahmen der betrieblichen Gesundheitsförderung (BGF) an.

## Lokale Ansätze zur Reduktion arbeitsbedingter Fatigue in der Anästhesiologie

### Fatigue-Risk-Management-Systeme

Um Fatigue-bedingte Fehler strukturell zu vermeiden, lohnt ein Blick auf andere kritische Branchen wie die Luftfahrt, die Fatigue Risk Management Systeme (FRMS) einsetzen. Diese Systeme bieten Ansätze, Fatigue zu erkennen und zu minimieren, und wurden auch von der ESAIC im Konsensusdokument über Nachhaltigkeit empfohlen [27].

Prädiktive Maßnahmen umfassen Dienstplananalysen, Alarmsysteme und die Überwachung von Arbeitszeiten [42, 45]. Proaktive Ansätze beinhalten die Meldung von Fatigue-Zuständen (z. B. per App), die Analyse des Schlafverhaltens vor Dienstbeginn, psychomotorische Tests sowie wachheitssteigernde Maßnahmen wie Bewegung oder Koffeinkonsum. Reaktive Maßnahmen schließen Reporting-Systeme, Schulungen und die Analyse besonders anfälliger Zeiten ein [7, 45].

FRMS behandeln Fatigue als strukturelles Problem

In Großbritannien werden partizipative Ansätze genutzt, bei denen Teams gemeinsam Strategien zur Problemlösung entwickeln. Diese Maßnahmen erzielen hohe Akzeptanz, da sie aus einem Konsens im Team resultieren. Anschließend erfolgt eine Erfolgskontrolle durch Datenerhebung und Überwachung [14, 45].

Im Gesundheitssystem stellt sich jedoch die Frage nach der praktischen Umsetzung solcher Systeme. Während ein Pilot bei erhöhtem Fatigue-Risiko einen Flug verweigern kann, steht diese Option bei nächtlichen Notfalloperationen nicht zur Verfügung. Ob in solchen Fällen eine Ablösung möglich ist, hängt von der lokalen Struktur und der Personalsituation ab [40].

Bei der Implementierung solcher Systeme sollte der Datenschutz berücksichtigt werden, da sie teilweise tiefgreifende Einschnitte mit sich führt.

### Warnsysteme

Aus unserem Alltag kennen wir bereits verschiedene Werkzeuge zur Bewertung von Fatigue, von denen einige auch in FRMS integriert werden könnten. Dazu zählen beispielsweise „Wearables“, die als digitale Uhren getragen werden und den Schlaf überwachen, oder psychomotorische Überwachungssysteme in Fahrzeugen, die fehleranfälliges Fahrverhalten erkennen. In einigen Branchen wird vor Dienstantritt ein „Fit-for-duty“-Test gefordert, um die Arbeitsfähigkeit sicherzustellen. Allerdings verfügen nur wenige dieser Tests über ausreichend hohe Sensitivität und Spezifität [1, 5, 11].

Digitale Unterstützung bietet auch bei der Dienstplanung eine Möglichkeit, vor erhöhtem Fatigue-Risiko zu warnen. Biomathematische Programme ermöglichen einen Überblick über das individuelle Risiko, berücksichtigen jedoch keine persönlichen Faktoren wie Schlafmangel vor Dienstbeginn [34, 51].

### Verhalten im Nachtdienst

Obwohl Mitarbeitende im Gesundheitswesen wissen, dass sie oft zu ungünstigen Zeiten arbeiten müssen, fehlt vielen das Wissen über den richtigen Umgang mit Nachtarbeit. Je nach Befragung haben 90–98,6 % der Teilnehmenden keine Schulung oder Hinweise dazu erhalten [14, 17]. Dabei wäre der Aufwand für solche Schulungen gering und könnte leicht in die Ausbildung integriert oder durch Pflichtfortbildungen ergänzt werden.

Schon in den 1980er-Jahren wurde nachgewiesen, dass ein 40-minütiges Nickerchen die Konzentration verbessert und Sekundenschlaf reduziert. Optimal ist ein Powernap von 10–20 min, um das Abrutschen in die Tiefschlafphase und kognitive Einschränkungen nach dem Aufwachen zu vermeiden. Alternativ kann ein vollständiger Schlafzyklus von ca. 90 min eingeplant werden [20, 31, 43]. Nach dem Dienst sollte man mindestens 7 h schlafen und auf gute Schlafbedingungen wie abgedunkelte Fenster achten [10, 47].

Während des Nachtdienstes sollte die Möglichkeit für einen „Powernap“ bestehen

Teamarbeit ist ein weiterer wichtiger Faktor: Sie ermöglicht Pausen für alle Teammitglieder und reduziert Fehler durch das Vieraugenprinzip. Regelmäßige Mahlzeiten zu festen Zeiten unterstützen die Verdauung, da diese dem zirkadianen Rhythmus folgt [47]. Auch regelmäßige körperliche Betätigung wirkt nachweislich gegen Fatigue [28].

### Dienstplanung und Arbeitsbelastung

Die Dienstplanung kann entscheidend dazu beitragen, Fatigue vorzubeugen. Nachtdienste dauern oft 9–13 h und sind damit länger als reguläre Tagesschichten, was zu einer höheren Belastung führt. Auch kurze Wechsel von Früh- zu Spätdiensten wirken ähnlich belastend wie Nachtdienste und sollten konsequent vermieden werden [25, 28].

In einer aktuellen europäischen Umfrage berichteten zwei Drittel der Anästhesist:innen von Problemen bei der Einhaltung der Ruhezeiten gemäß der European Working Time Directive. Ebenso viele gaben an, mehr zu arbeiten als vertraglich vereinbart. Diese Überbelastung erhöht das Risiko für Fatigue [13].

Hinzu kommt, dass aus organisatorischen Gründen häufig elektive Patient:innen im Bereitschaftsdienst operiert werden, wodurch die eigentliche Bereitschaftszeit zunehmend im OP verbracht wird [17]. Hier ist es Aufgabe des OP-Managements, sicherzustellen, dass der Bereitschaftsdienst nicht zur Regelarbeitszeit wird. Beispielsweise sollten am Tage ankommende, nachgemeldete Patient:innen bereits in einem Notfall-OP vom Tagdienst behandelt werden.

## Europäische Strategien und Regulierungen zur arbeitsbedingten Fatigue

Ein zentraler Akteur auf internationaler Ebene ist das WWW des EBA, dem eine der Autor:innen angehört. 2019 wurde dort das Fatigue Project gestartet, mit dem Ziel, Daten zur arbeitsbedingten Fatigue europäischer Anästhesist:innen zu sammeln und publik zu machen. Ziele sind es, das Bewusstsein für die Auswirkungen auf Patientensicherheit und Wohlbefinden zu schärfen sowie eine Veränderung der Kultur und Vorschriften voranzutreiben – von einzelnen Anästhesist:innen über Krankenhausleitungen bis hin zu Regulierungsbehörden.

Das EBA appelliert an Fachkräfte im Gesundheitswesen, sich aktiv für bessere Bedingungen einzusetzen. Nationale Organisationen werden gezielt angesprochen, um die Kampagne zu unterstützen und die Einführung von FRMS europaweit zu fördern – auch in anderen medizinischen Disziplinen. Darüber hinaus wird auf eine Reform der Gesundheitsvorschriften auf europäischer Ebene hingearbeitet. Veröffentlichungen aus der Arbeitsgruppe des WWW sind Teil dieses Reviews [23, 52].

Neben der wissenschaftlichen Arbeit wurde seit Beginn des Projekts auch ein starker Fokus auf edukative Ansätze gelegt. Arbeitsbedingte Fatigue wurde auf europäischen und nationalen Kongressen thematisiert, um das Problembewusstsein zu schärfen [23, 52].

Die European Patient Safety Foundation (EPSF) nahm das Momentum auf, das 2015 in Großbritannien nach dem Tod eines Trainees entstanden ist. In der „Joint Fatigue Group“ koordiniert die EPSF ihre Arbeit mit der Association of Anaesthetists, dem Royal College of Anaesthetists und der Faculty of Intensive Care Medicine, oft auch in Zusammenarbeit mit dem EBA oder der European Society of Anaesthesiology [21].

Aktuell hat die EPSF einen Antrag auf europäische Kooperation (COST) gestellt, der von einer der Autor:innen unterstützt wird. Sollte er bewilligt werden, könnten über 4 Jahre verschiedene Arbeitsgruppen gefördert werden.

Das britische Gesundheitssystem ist in puncto Fatigue-Management vielen Ländern voraus, was auf die hohe Institutionalisierung und frühzeitigen Bestrebungen zur Aufarbeitung des Themas zurückzuführen ist. Bereits implementierte FRMS und ein kürzlich veröffentlichtes Weißbuch mit Empfehlungen zur erfolgreichen Umsetzung zeigen die Fortschritte [14].

## Fazit für die Praxis

- Arbeitsbedingte Fatigue stellt ein bedeutendes strukturelles Problem im Gesundheitssystem dar.
- Sie gefährdet nicht nur die Patientensicherheit, sondern auch die Gesundheit der Ärzt:innen.
- Fatigue betrifft Fachkräfte auf allen Karrierestufen, wobei die zugrunde liegenden Faktoren variieren.
- Angesichts einer zunehmenden Lebensarbeitszeit wird es für Arbeitgeber:innen unerlässlich, sich im Rahmen einer nachhaltigen Personalführung gezielt mit diesem Thema auseinanderzusetzen.
- Eine wirksame Maßnahme, die auf europäischer Ebene zunehmend gefordert und in einigen Ländern bereits teilweise umgesetzt wird, ist die Einführung von *Fatigue-Risk-Management-Systemen.*
